# Giant cell myocarditis attributable to myositis: therapeutic management under the guidance of serial endomyocardial biopsy—a case report

**DOI:** 10.1093/ehjcr/ytae326

**Published:** 2024-07-10

**Authors:** Marina Arai, Yu Kataoka, Yasumasa Tsukamoto, Keiko Ohta-Ogo

**Affiliations:** Department of Cardiovascular Medicine, National Cerebral and Cardiovascular Center, 6-1 Kishibeshinmachi, Suita, Osaka 564-8565, Japan; Department of Transplant Medicine, National Cerebral and Cardiovascular Center, Suita, Japan; Department of Cardiovascular Medicine, Graduate School of Medicine, Tohoku University, Sendai, Japan; Department of Cardiovascular Medicine, National Cerebral and Cardiovascular Center, 6-1 Kishibeshinmachi, Suita, Osaka 564-8565, Japan; Department of Transplant Medicine, National Cerebral and Cardiovascular Center, Suita, Japan; Department of Pathology, National Cerebral and Cardiovascular Center, Suita, Japan

**Keywords:** Case report, Giant cell myocarditis, Giant cell myositis, Endomyocardial biopsy, Immunosuppressive therapy

## Abstract

**Background:**

Giant cell myocarditis is a fatal disease that could be rapidly progressive if not properly managed. However, the role of immunosuppressive therapy, especially in refractory cases, remains unclear.

**Case summary:**

A 76-year-old man presented with back pain with elevated cardiac enzymes. Skeletal muscle and endomyocardial biopsies revealed giant cell myositis and giant cell myocarditis. Despite the initial immunosuppressive therapy, cardiac enzymes continued to rise. Serial endomyocardial biopsies enabled combination treatment of prednisolone, cyclosporine, and mycophenolate mofetil according to histological inflammatory activity.

**Discussion:**

We presented a case of refractory giant cell myocarditis preceded by giant cell myositis. While endomyocardial biopsy is an approach with risk of procedural complications, it can guide giant cell myocarditis management when the initial immunosuppressive therapy is ineffective.

**Learning points**

- Giant cell myocarditis could present the concomitance of giant cell myositis, which causes a variety of cardiac and non-cardiac symptoms.
- Serial endomyocardial biopsy could guide the selection of appropriate therapies in a case with giant cell myocarditis refractory to ongoing management.
- Disease activity of giant cell myocarditis may be evaluable by serial evaluation of both cardiac enzymes and myocardial biopsy specimens.

## Introduction

Giant cell myocarditis (GCM) is a rare autoimmune disease that could be rapidly progressive and fatal if not properly managed. Although therapeutic guideline is not established, several studies reported the benefit of prednisolone and cyclosporine combination therapy. However, some GCM cases exhibit poor response to this combination approach, and it remains unclear how to select additional agent in such refractory cases.

## Summary figure

**Figure:**
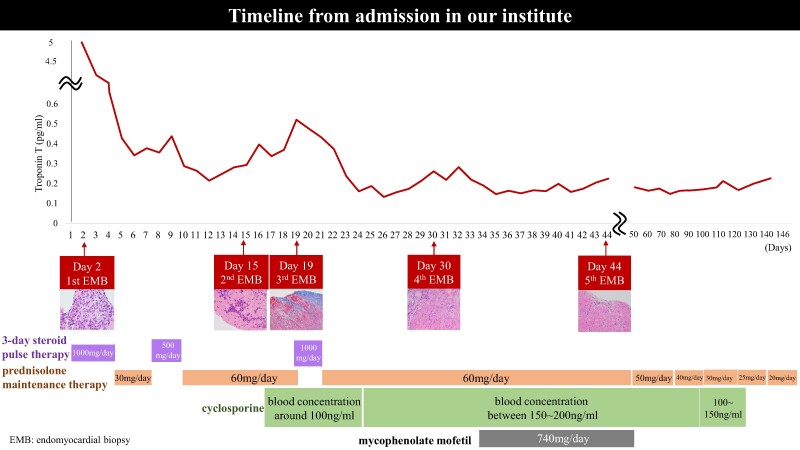


## Case presentation

**Figure 1:**
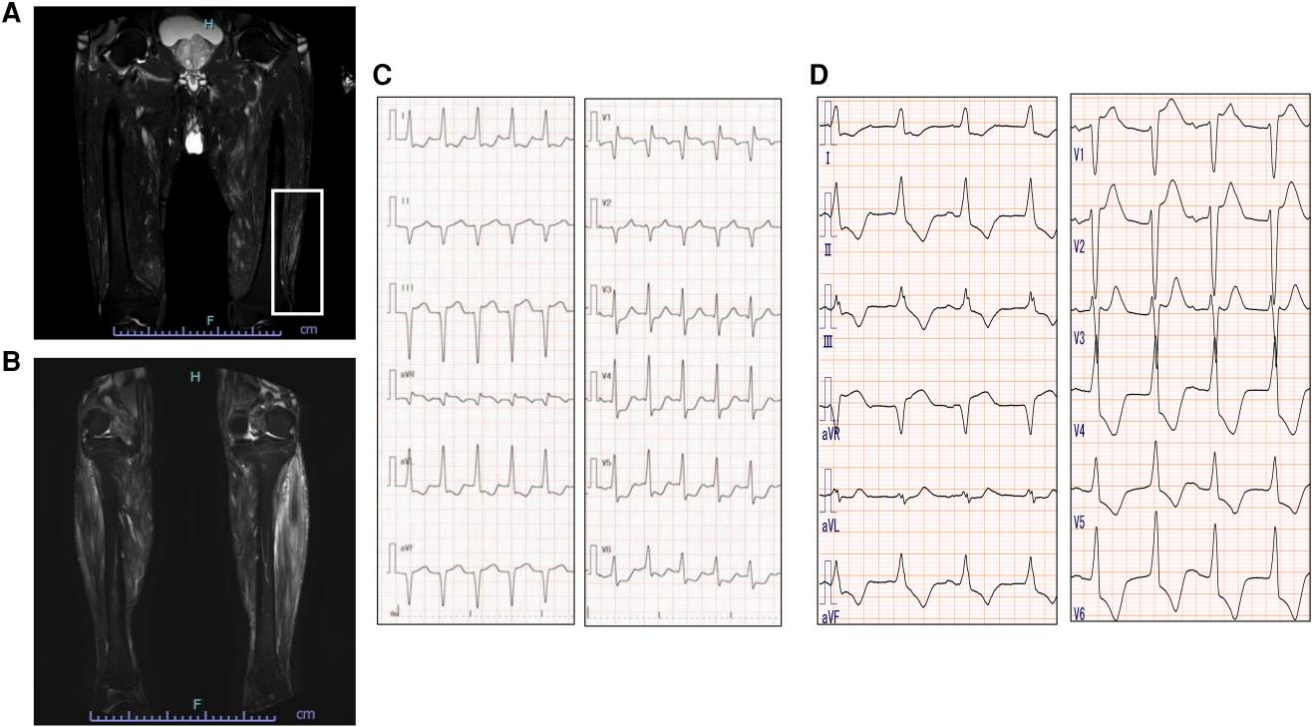
T_2_-weighted magnetic resonance imaging of the lower limb and electrocardiogram. Multiple lesions with high signal intensities were observed in both the proximal (*A*, yellow rectangle) and distal muscles including anterior tibial muscles and gastrocnemius (*B*). The initial electrocardiogram showed sinus tachycardia (*C*). Then, he exhibited complete atrioventricular block (*D*).

A 76-year-old gentleman had a history of diabetes and hypertension. He was treated with 25 mg sitagliptin and 5 mg amlodipine. He did not have smoking/drinking habits, mental illness, disability, and any history of substance abuse. He did not have any family history of autoimmune disease. He was hospitalized in the local hospital due to his low back pain and myalgia of bilateral lower legs. On T_2_-weighted magnetic resonance imaging (MRI), multiple lesions with high signal intensities were observed extensively in the back and the lower limb muscles (*Figure 1A* and *B*). Since he presented hypoxia and elevated cardiac enzymes after hospitalization, he was transferred to the cardiovascular care unit at our national heart institute.

**Figure 2:**
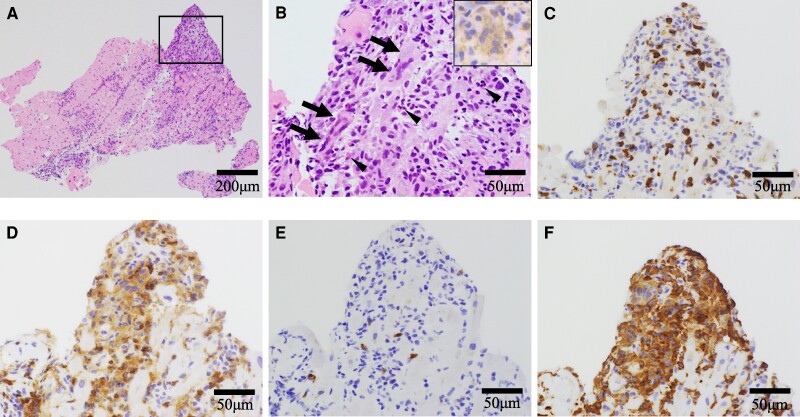
Histopathology of the first endomyocardial biopsy. The first endomyocardial biopsy demonstrated severe inflammatory cell infiltration (*A* and *B*). Giant cells (arrows) and eosinophils (arrowheads) existed. The inset in (*B*) showed the presence of CD68-positive cells. Immunohistochemistry showed infiltration of T lymphocyte (*C*, CD3) with predominant CD4-positive cells, representing helper T cells (*D*) rather than CD8-positive cells (*E*). Abundant CD168-positive cells reflecting M2 macrophages were also observed (*F*).

**Figure 3:**
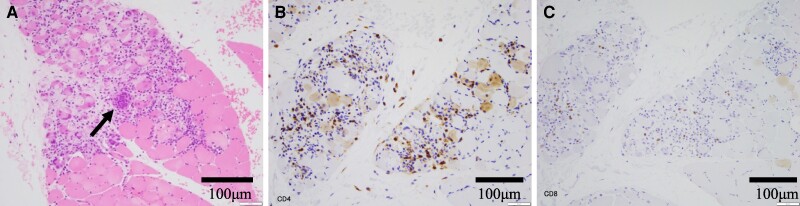
Histopathology of the skeletal muscle biopsy. Haematoxylin and eosin staining identified giant cells (arrow), lymphocytes, and eosinophils (*A*). Immunohistochemistry showed predominant CD4-positive cells (*B*) over CD8-positive cells (*C*), which were like those in myocardium.

His heart sound was normal without any murmurs. He had moist rales in both lungs and general malaysia. The initial electrocardiogram showed sinus tachycardia with ST-segment depression (lead I, aVL, and V3–V6) and elevation in lead aVR (*Figure 1C*). Then, his cardiac rhythm abruptly changed to complete atrioventricular block (*Figure 1D*). His biochemistry data demonstrated a high troponin T (TnT = 3.35 pg/mL) level. Additionally, echocardiography showed mildly reduced left ventricular ejection fraction with its increased wall thickness (see Supplementary material online, *Video S1*). Emergent coronary angiography identified one moderate stenosis in his right coronary artery (see Supplementary material online, *Videos S2* and *S3*), and fractional flow reserve of this lesion was 0.99. We conducted endomyocardial and the left lateral vastus muscle biopsies. The histopathology of initial endomyocardial biopsy (EMB) revealed severe inflammatory cell infiltration with the presence of multinucleated giant cells, lymphocytes, macrophages, and eosinophils with myocyte necrosis (*Figure 2*). He was pathologically diagnosed as GCM. His skeletal muscle biopsy showed similar results, suggesting active giant cell myositis (*Figure 3*). All measured autoantibodies and tumour markers except anti-striated muscle and anti-titin antibodies were negative in this case (see Supplementary material online, *Table S1*). Computed tomography imaging did not identify any suggestive features of thymoma and other tumours.

**Figure 4:**
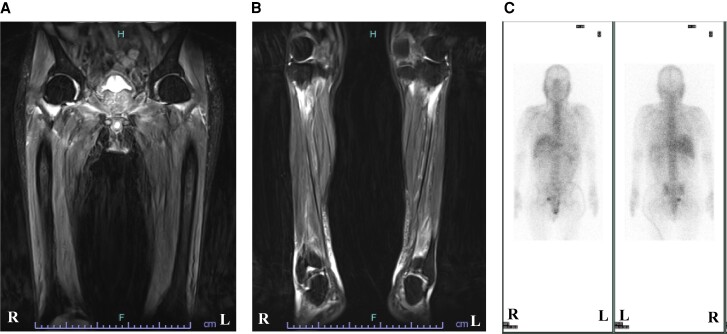
Follow-up T_2_-weighted magnetic resonance imaging of the lower limbs and gallium scintigraphy. The follow-up magnetic resonance imaging on Day 38 revealed the absence of high signal intensity lesions in both the proximal (*A*) and distal muscles (*B*). Gallium scintigraphy on Day 45 did not show any visible accumulation of gallium (*C*).

**Figure 5:**
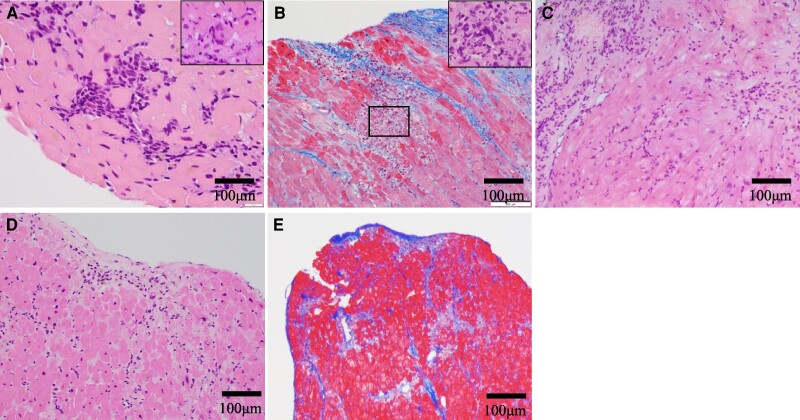
Serial histopathological changes of follow-up endomyocardial biopsies. The second endomyocardial biopsy (*A*). Haematoxylin and eosin staining revealed focal inflammation with a few giant cells (inset). The third endomyocardial biopsy (*B*). Reparative fibrotic changes with a focus of myocyte injury were demonstrated by Masson’s trichrome staining. The inset presented haematoxylin and eosin staining. The fourth endomyocardial biopsy (*C*). Fibrotic replacement was further spreading, but inflammation still existed. The fifth endomyocardial biopsy (*D* and *E*). Haematoxylin and eosin staining identified that reparative changes mainly occurred and multinucleated giant cells disappeared (*D*). Reparative of fibrosis was observed by Masson’s trichrome staining (*E*).

Three-day methylprednisolone pulse therapy (1000 mg/day) was commenced, followed by 30 mg prednisolone. However, TnT gradually increased to 0.377 ng/mL with atrial tachycardia on Day 7. Therefore, 3-day methylprednisolone pulse therapy (500 mg/day) and the escalated dose of prednisolone (60 mg) were commenced. Furthermore, cyclosporine was added with plasma trough concentration maintained at around 100 ng/mL. Troponin T level was transiently lowered by 0.214 ng/mL after these therapies. On Day 15, TnT level re-elevated, suggesting inflammation refractory to the initial immunotherapies. As such, a second EMB was performed on that day. On Day 15, TnT level re-elevated, and it was considered to present an inflammatory status refractory to the therapies, and then the second EMB was performed on the day. It revealed reparative change with reduced inflammatory cell infiltration, although there were still small areas exhibiting inflammatory activity (*Figure 4A*). Based on these findings, we continued same regimens. Because TnT level re-elevated again from Day 17, the third EMB was performed on Day 19. It showed that inflammatory focus with myocardial injury still existed (*Figure 4B*). Therefore, the third 3-day methylprednisolone pulse therapy (1000 mg/day) was used again with the control of plasma trough concentration of cyclosporin between 150 and 200 ng/mL. Despite these intensified therapies, a continuing increase in TnT level was observed. The fourth EMB on Day 30 still demonstrated ongoing inflammation and fibrotic process (*Figure 4C*), and then mycophenolate mofetil was added to further modulate inflammatory activity. Following this regimen, TnT level did not further increase. The T_2_-weighted MRI of his lower limbs on Day 38 revealed a disappearance of high signal intensities (*Figure 4A* and *B*). The fifth EMB on Day 44 showed mainly reparative changes (*Figure 5D* and *E*). Furthermore, gallium scintigraphy on Day 45 did not identify any abnormal inflammatory signals (*Figure 4C*). Mycophenolate mofetil was discontinued on Day 48 due to the occurrence of pancytopenia. After the discontinuation of mycophenolate mofetil, pancytopenia was dissolved. Echocardiography on Day 67 revealed normal wall thickness of his left ventricle returned (see Supplementary material online, *Video S4*). However, after Day 88, renal function declined potentially due to cyclosporin. In addition, he had candidemia on Day 90. We decided to discontinue cyclosporin on Day 128. He was deceased on Day 146 due to sepsis of candidemia.

## Discussion

Giant cell myocarditis is a rare disease, and around 20% of GCM has been shown to concomitantly present other autoimmune disease.^1,2^ Our case presented giant cell myositis, in addition to GCM, which causes a variety of cardiac and non-cardiac symptoms. Given that both skeletal biopsy and EMB revealed the presence of giant cell, this clinical course indicates systematic involvement of giant cell-related inflammation.

In our case, repeated EMB helped to evaluate response of myocardium to immunosuppressive therapies. While EMB has a risk of procedural complications, it enables to evaluate how inflammatory activity is altered by therapeutic management. Serial EMBs could help to guide immunosuppressive therapies of GCM when the initial management does not respond well.

Cardiac MRI was not conducted due to unstable condition after hospitalization. Since EMB is an invasive testing for risk stratification and prognostication,^3^ cardiac MRI could be considered as an alternative approach to non-invasively evaluate tissue-level pathologies.

The combination of corticosteroids with one or two additional immunosuppressive agents is generally used in patients with GCM.^1–3^ Our case was refractory to the use of corticosteroid and cyclosporine. Since one recent case report showed successful management with corticosteroid, cyclosporine, and mycophenolate mofetil in a patient presenting recurrent GCM,^2,4^ we selected these triple combination therapy in our GCM case. Considering that serial EBM examination revealed the improvement of inflammatory activity following the use of mycophenolate mofetil, this combination could be one of effective therapeutic regimens to manage refractory GCM.

While intravenous immunoglobulin may have a potential to modulate disease activity in GCM, its clinical efficacy in fulminant myocarditis has not been fully demonstrated yet.^5–7^ Further investigation is required to investigate whether intravenous immunoglobulin is effective in patients with GCM.

We experienced a case of GCM preceded by giant cell myositis. While the initial immunosuppressive therapy was ineffective, combination of prednisolone, cyclosporine, and mycophenolate mofetil under the guidance of serial EMBs successfully controlled disease activity. Our case may underscore serial EMB-based management in a patient with both GCM and giant cell myositis refractory to immunosuppressive therapy.

## Supplementary Material

ytae326_Supplementary_Data

## Data Availability

The data underlying this article will be shared upon reasonable request to the corresponding author.

## Lead author biography

Marina Arai is a cardiologist at the National Cerebral and Cardiovascular Center in Osaka, Japan, and a fourth-year graduate student at Tohoku University in Sendai, Japan.
