# Supplementary material for: Giant cell myocarditis attributable to myositis: therapeutic management under the guidance of serial endomyocardial biopsy—a case report
Source: Eur Heart J Case Rep. 2024 Jul 10;8(7):ytae326. doi: 10.1093/ehjcr/ytae326 (PMC11263867; doi:10.1093/ehjcr/ytae326)
Supplement: ytae326_Supplementary_Data [file ytae326_supplementary_data.zip › Supplemental Appendix.docx]

**Supplementary-Table Legend**

**Table S1. Measured Autoantibodies and Tumor Makers in This Case**

All of measured autoantibodies and tumor markers except anti-striated muscle antibody and anti-titin antibody were negative in this case.

ARS=amino acyl-tRNA synthetases, CA=carbohydrate antigen, CEA=carcinoembryonic antigen, MPO-ANCA=myeloperoxidase anti-neutrophil cytoplasmic antibody, PR3 ANCA= proteeinase3 anti-neutrophil cytoplasmic antibody, RNP=ribonucleoprotein, SRP=signal recognition particle

**Supplementary-Video Legends**

**Video S1. Echocardiography on Admission**

Echocardiography on admission identified an increased left ventricular wall thickness (=10mm), which indicated interstitial edema of left ventricular wall. left ventricular ejection fraction was about 40%. There were no obvious regional wall motion abnormalities and pericardial effusion.

**Video S2. Left Coronary Angiography on Admission**

There was severe stenosis in the diagonal branch of his left coronary artery.

**Video S3. Right Coronary Angiography on Admission**

There were two moderate stenosis in the proximal and distal segments of his right coronary artery. Fractional flow reserve was 0.99.

**Video S4. Echocardiography on Day 67**

The wall thickness of his left ventricle returned to normal (7-8mm).
