# Supplementary material for: Giant cell myocarditis attributable to myositis: therapeutic management under the guidance of serial endomyocardial biopsy—a case report
Source: Eur Heart J Case Rep. 2024 Jul 10;8(7):ytae326. doi: 10.1093/ehjcr/ytae326 (PMC11263867; doi:10.1093/ehjcr/ytae326)
Supplement: ytae326_Supplementary_Data [file ytae326_supplementary_data.zip › Table S1.docx]

| **Autoantibodies** | |
| --- | --- |
| Antinuclear antibody | < 40 times |
| PR3-ANCA | < 1.0 U/ml |
| MPO-ANCA | < 1.0 U/ml |
| Anti-RNP antibody | < 2.0 U/ml |
| Anti-Jo1 antibody | < 1.0 U/ml |
| Anti-ARS antibody | < 5.0 |
| Anti-Acetylcholine receptor antibody | 0.2 |
| Anti-mitochondria antibody | < 1.5 |
| Anti-striated muscle antibody | positive |
| Anti-titin antibody | 0.71 |
| Anti-SRP antibody | < 1.0 U/ml |
| **Tumor makers** | |
| CEA | 2.2 ng/ml |
| CA19-9 | 11.8 U/ml |
| CA125 | 21.6 U/ml |

**Supplementary-Table. Measured Autoantibodies and Tumor Makers in This Case**
